# Tuberculous Abdominal Cocoon

**DOI:** 10.4269/ajtmh.2011.10-0620

**Published:** 2011-01-05

**Authors:** Ankur Gadodia, Raju Sharma, Nadarajah Jeyaseelan

**Affiliations:** Department of Radio-diagnosis All India Institute of Medical Sciences, New Delhi, India

A 22-year-old male patient presented with the complaints of upper abdominal colicky pain, intermittent bilious vomiting, and abdominal distension of 3-month duration. There was no history of peritonitis, abdominal surgery, or long-term medication. Clinical examination was unremarkable. Routine laboratory investigations revealed hemoglobin of 11 gm/dL, erythrocyte sedimentation rate (ESR) of 80 min the first hour and normal leukocyte count, platelets, electrolytes, renal, and liver function tests. Chest and abdominal radiograph were normal. Clinical diagnosis of subacute intestinal obstruction was made. Barium meal follow through (BMFT) revealed adherent small bowel loops with delayed transit time (Figure 1). Mucosal pattern and illeoceacal junction were normal. Multi-detector computed tomography (MDCT) of the abdomen showed a 3-mm-thick membrane encasing the small bowel loops and forming a saclike structure (Figure 2A). Sagittal and coronal reformation better demonstrated the entire disease process (Figure 2B). Omental thickening, retroperitoneal adenopathy, and free pelvic fluid were also demonstrated. Ultrasound-guided fine needle aspiration cytology (FNAC) of the omentum revealed caseating granuloma and giant cells. After surgery a post-operative histology confirmed diagnosis of tuberculosis.

**Figure 1.**
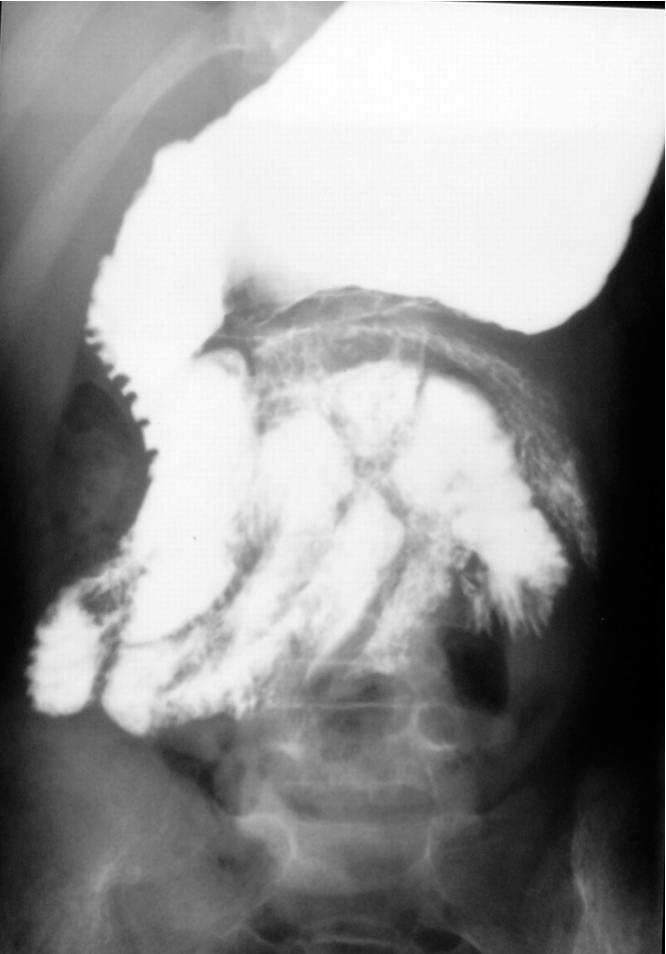
BMFT image shows adherent small bowel loops clumped in the central abdomen.

**Figure 2.**
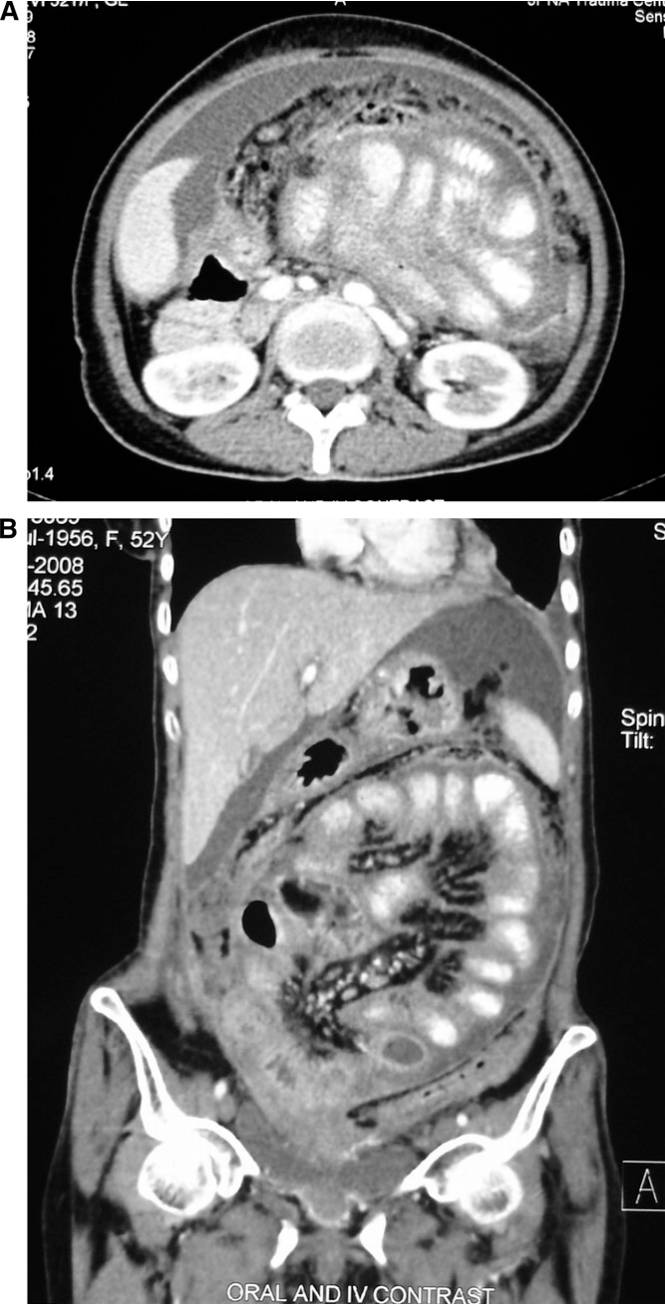
Multi-detector computed tomography (MDCT) of the abdomen axial (**A**) and coronal reformatted (**B**) images reveal a 3-mm-thick membrane encasing the small bowel loops and forming a saclike structure.

Described by Foo and others,^1^ abdominal cocoon is a rare cause of intestinal obstruction characterized by encasement of the small bowel by a thick, fibrous membrane. Sclerosing encapsulating peritonitis (SEP) also known as abdominal cocoon primarily affects adolescent females. The SEP can be classified as idiopathic or secondary to long-term peritoneal dialysis, beta-blocker practolol intake, orthotopic liver transplantation, abdominal surgery, sarcoidosis, systemic lupus erythematosus, gastrointestinal malignancy, and abdominal tuberculosis.^2^ Abdominal cocoon secondary to tuberculosis is rare with few reported cases.^3^,^4^ Clinical manifestations of abdominal cocoon are non-specific and include intestinal obstruction and/or abdominal mass.^1^–^5^ A majority of the reported cases were diagnosed incidentally at laparotomy. Preoperative diagnosis of abdominal cocoon is difficult because of non-specific clinical features and reduced awareness. Imaging (barium meal follow through and CT) studies play an important role in the definitive preoperative diagnosis of abdominal cocoon. Radiographs are not specific and may show evidence of small bowel obstruction. The BMFT shows reduced transit time and serpentine or concertina-like configuration of dilated small bowel loops in a fixed U-shaped cluster. The CT best shows the fibrous membrane encasing the bowel loops; thus, helps in reaching a definite diagnosis. Recent reports have emphasized the role of MDCT with saggital and coronal reconstructions in demonstrating the extent of disease for surgical planning and in showing subtle radiological findings.^6^,^7^

## References

[1] Foo KT Ng KC Rauff A Foong WC Sinniah R 1978 Unusual small intestinal obstruction in adolescent girls: the abdominal cocoon Br J Surg 65 427 430 656764 10.1002/bjs.1800650617

[2] Ibrahim NA Oludara MA 2009 Abdominal cocoon in an adolescent male patient Trop Doct 39 254 256 19762590 10.1258/td.2009.090104

[3] Basu A Sukumar R Sistla SC Jagdish S 2007 “Idiopathic” abdominal coccon Surgery 141 277 278 17299859 10.1016/j.surg.2005.12.004

[4] Lalloo S Krishna D Maharajh J 2002 Case report: abdominal cocoon associated with tuberculous pelvic inflammatory disease Br J Radiol 75 174 176 11893642 10.1259/bjr.75.890.750174

[5] Hur J Kim KW Park MS Yu JS 2004 Abdominal cocoon: preoperative diagnostic clues from radiologic imaging with pathologic correlation AJR Am J Roentgenol 182 639 641 14975962 10.2214/ajr.182.3.1820639

[6] Tombak MC Apaydin FD Colak T Duce MN Balci Y Yazici M Kara E 2010 An unusual cause of intestinal obstruction: abdominal cocoon AJR Am J Roentgenol 194 W176 178 20093570 10.2214/AJR.09.3083

[7] Wang Q Wang D 2010 Abdominal cocoon: multi-detector row CT with multiplanar reformation and review of literatures Abdom Imaging 35 92 94 19048332 10.1007/s00261-008-9489-0

